# Matroidal Structure of Generalized Rough Sets Based on Tolerance Relations

**DOI:** 10.1155/2014/243070

**Published:** 2014-08-05

**Authors:** Hui Li, Yanfang Liu, William Zhu

**Affiliations:** Laboratory of Granular Computing, Minnan Normal University, Zhangzhou 363000, China

## Abstract

Rough set theory provides an effective tool to deal with uncertain, granular, and incomplete knowledge in information systems. Matroid theory generalizes the linear independence in vector spaces and has many applications in diverse fields, such as combinatorial optimization and rough sets. In this paper, we construct a matroidal structure of the generalized rough set based on a tolerance relation. First, a family of sets are constructed through the lower approximation of a tolerance relation and they are proved to satisfy the circuit axioms of matroids. Thus we establish a matroid with the family of sets as its circuits. Second, we study the properties of the matroid including the base and the rank function. Moreover, we investigate the relationship between the upper approximation operator based on a tolerance relation and the closure operator of the matroid induced by the tolerance relation. Finally, from a tolerance relation, we can get a matroid of the generalized rough set based on the tolerance relation. The matroid can also induce a new relation. We investigate the connection between the original tolerance relation and the induced relation.

## 1. Introduction

Rough set theory was originally proposed by Pawlak [1, 2] in 1982 and serves as a new mathematical approach to vague concept. It has been widely applied to many different fields, such as knowledge discovery [3], machine learning [4], knowledge acquisition [5], decision analysis [6, 7], and granular computing [8]. It is well known that the classical rough set theory is based on equivalence relations. However, equivalence relations are restrictive for many applications. To address this problem, classical rough set theory has been extended from equivalence relations to some other relations, such as tolerance relation [9, 10], similarity relation [11, 12], and arbitrary relation [13–15].

Matroid theory [16, 17] proposed by Whitney is a generalization of both linear algebra and graph theory. It has been successfully applied to various fields, such as combinatorial optimization, algorithm design, information coding, and cryptology. In order to enrich the theoretical system and extend the applications of rough sets, it is helpful to study rough sets with matroids. There are many works [18–31] about the connection between matroids and rough sets.

From a tolerance relation, a matroidal structure is proposed in this paper. First, we define a family of sets through the lower approximation based on a tolerance relation and prove the family to satisfy the circuit axioms of matroids. Hence, we obtain a matroid with the family of sets as its circuits. Moreover, the family of sets are proved to be a partition, so the matroid is a partition-circuit matroid. Second, we obtain that the family of circuits of this matroid is equal to the partition induced by the transitive closure of the tolerance relation. Next we investigate some characteristics of this matroid through the generalized rough set based on a tolerance relation, such as the base and the rank function. Third, we study some important relationships between the closure operator of this matroid and the upper approximation operator of the tolerance relation. Finally, we know that the matroid established by a tolerance relation can induce a relation. We prove that the original tolerance relation is contained in the induced relation. In particular, the induced relation is equal to the transitive closure of the original tolerance relation.

The rest of this paper is organized as follows.  Section 2 reviews some fundamental definitions and properties of generalized rough sets and matroids. In Section 3, we propose a matroid induced by a tolerance relation and study some characteristics of this matroid through generalized rough sets. Then we investigate the relationship between the closure operator of this matroid and the upper approximation operator of a tolerance relation. We also study the relationship between a tolerance relation and the relation induced by the matroid established by the original tolerance relation. We conclude this paper in Section 4.

## 2. Basic Definitions

In this section, we recall some fundamental definitions and important conclusions of generalized rough sets and matroids.

### 2.1. Rough Set

Let *U* be a universe, *U* × *U* the product set of *U* and *U*. Any subset *R* of *U* × *U* is called a binary relation on *U*. For any (*x*, *y*) ∈ *U* × *U*, if (*x*, *y*) ∈ *R*, we say *x* has relation *R* with *y* and denote this relationship as *xRy*. In the rest of this paper, we assume *U* is a finite and nonempty set unless otherwise stated.

In rough sets, a pair of approximation operators are used to describe an object. In the following definition, we introduce the lower and upper approximation operators of generalized rough sets through the neighborhood.

**Definition 1 (lower and upper approximation operators [32]).**

Let *R* be a relation on *U*. A pair of operators $\underline{R}$, $\bar{R}:2^{U}\to 2^{U}$ are defined as follows: for all *X*⊆*U*,

(1) R_(X)={x∈U:RN(x)⊆X},R¯(X)={x∈U:RN(x)∩X≠∅},

where *RN*(*x*) = {*y* ∈ *U* : *xRy*} is called the neighborhood of *x* with respect to *R*. $\underline{R}$, $\bar{R}$ are called the lower and upper approximation operators, respectively.

The following proposition presents some properties of lower approximation operator.

**Proposition 2 (see [32]).**

Let *R* be a relation on *U*. $\underline{R}$ satisfies the following properties: for all *X*, *Y*⊆*U*,

1. $\underline{R}(U)=U$;
2. $\underline{R}(X\cap Y)=\underline{R}(X)\cap \underline{R}(Y)$;
3. $\underline{R}(X\cup Y)⊇\underline{R}(X)\cup \underline{R}(Y)$;
4. $X\subseteq Y\Rightarrow \underline{R}(X)\subseteq \underline{R}(Y)$.

We give the definition of tolerance relation, a special type of relation.

**Definition 3 (tolerance relation [10, 33]).**

Let *R* be a relation on *U*. If, for any *x* ∈ *U*, *x* ∈ *RN*(*x*), *R* is reflexive. If, for any *x*, *y* ∈ *U*, *y* ∈ *RN*(*x*)⇒*x* ∈ *RN*(*y*), one says *R* is symmetric. If *R* is both reflexive and symmetric, *R* is called a tolerance relation.

The following results hold for reflexive and symmetric relations.

**Proposition 4 (see [32]).**

Let *R* be a relation on *U*. For all *X*⊆*U*,

1. *R* is reflexive $\Leftrightarrow \underline{R}(X)\subseteq X\Leftrightarrow X\subseteq \bar{R}(X)$;
2. *R* is symmetric $\Leftrightarrow \;X\subseteq \underline{R}(\bar{R}(X))\Leftrightarrow \bar{R}(\underline{R}(X))\subseteq X$.

### 2.2. Matroid

There are many equivalent ways to define a matroid. The following definition of matroid is presented from the viewpoint of independent sets.

**Definition 5 (matroid [16]).**

A matroid is an ordered pair *M* = (*U*, *I*) consisting of *U* and a collection *I* (called independent sets) of subsets of *U* with the following three properties:

(I1) *∅* ∈ *I*;
(I2) If *I* ∈ *I* and *I*′⊆*I*, then *I*′ ∈ *I*;
(I3) If *I*_1_, *I*_2_ ∈ *I* and |*I*_1_| < |*I*_2_|, then there exists *e* ∈ *I*_2_ − *I*_1_ such that *I*_1_⋃{*e*} ∈ *I*, where |*I*_1_| denotes the cardinality of *I*_1_.

**Example 6 .**

Let *U* = {*a*, *b*, *c*, *d*}, *I* = {*∅*,  {*a*},  {*b*},  {*c*},  {*d*},  {*a*, *b*},  {*a*, *c*},  {*b*, *c*},  {*b*, *d*},  {*a*, *d*},  {*c*, *d*},  {*a*, *b*, *c*},  {*a*, *b*, *d*},  {*b*, *c*, *d*}}. Then *M* = (*U*, *I*) is a matroid.

In order to make some expressions clear and brief, we introduce some symbols as follows.

**Definition 7 (see [16]).**

Let *U* be a universe. *A*⊆2^*U*^ is a family of subsets of *U*; then

(2) Upp(A)={X⊆U:∃A∈A  s.t.  A⊆X};Max⁡(A)={X∈A:∀Y∈A,  X⊆Y⟹X=Y};Min⁡(A)={X∈A:∀Y∈A,  Y⊆X⟹X=Y};Opp(A)={X⊆U:X∉A}.

Base is an important concept of matroids. We give the definition of base as follows.

**Definition 8 (base [16]).**

Let *M* = (*U*, *I*) be a matroid. Any maximal independent set in *M* is called a base of *M* and the family of all bases of *M* is denoted by *B*(*M*); that is, *B*(*M*) = *Max*⁡(*I*).

If a subset of the universe is not an independent set of a matroid, it is called a dependent set of the matroid.

**Definition 9 (circuit [16]).**

Let *M* = (*U*, *I*) be a matroid. A minimal dependent set in *M* is called a circuit of *M* and one denotes the family of all circuits of *M* by *C*(*M*); that is, *C*(*M*) = *Min*⁡(Opp(*I*)).

The following proposition shows that a matroid can be defined from the viewpoint of circuits.

**Proposition 10 (circuit axioms [16]).**

Let *C* be a family of subsets of *U*. Then there exists *M* = (*U*, *I*) such that *C* = *C*(*M*) if and only if *C* satisfies the following conditions:

(C1) *∅* ∉ *C*;
(C2) If *C*_1_, *C*_2_ ∈ *C* and *C*_1_⊆*C*_2_, then *C*_1_ = *C*_2_;
(C3) If *C*_1_, *C*_2_ ∈ *C*, *C*_1_ ≠ *C*_2_, and *e* ∈ *C*_1_∩*C*_2_, then there exists *C*_3_ ∈ *C* such that *C*_3_⊆*C*_1_ ∪ *C*_2_ − {*e*}.

In matroid theory, the rank function serves as a quantitative tool. The definition of rank function is introduced as follows.

**Definition 11 (rank function [16]).**

Let *M* = (*U*, *I*) be a matroid. Then *r*_*M*_ is called the rank function of *M*, where *r*_*M*_(*X*) = max⁡⁡{|*I*| : *I*⊆*X*, *I* ∈ *I*} for all *X*⊆*U*. When there is no confusion, one omits the subscript *M*.

The following proposition shows the connection between the independent set and the rank function of a matroid.

**Proposition 12 (see [16]).**

Let *M* = (*U*, *I*) be a matroid and *r*_*M*_ its rank function. For all *X*⊆*U*, *r*_*M*_(*X*) = |*X*| if and only if *X* ∈ *I*.

The closure operator is one of the important characteristics of a matroid. We give the definition of closure operator as follows.

**Definition 13 (closure operator [16]).**

Let *M* = (*U*, *I*) be a matroid. For all *X*⊆*U*,

(3) cl⁡M(X) =X∪{e∈U−X:∃C∈C  such  that  e∈C⊆X∪{e}}

is called the closure of *X* in *M* and cl⁡_*M*_ is called the closure operator. One can omit the subscript *M* when there is no confusion.

## 3. Matroidal Structure Induced by Tolerance Relation

In this section, we establish a matroidal structure of the generalized rough set based on a tolerance relation. Firstly, a family of sets are defined and they are proved to satisfy the circuit axioms of matroids.

**Definition 14 .**

Let *R* be a tolerance relation on *U*. One defines a family of sets with respect to *R* as follows:

(4) $$\begin{array}{c} C\left(R\right)=\mathrm{Min}\left(\left\{X\subseteq U:\underline{R}\left(X\right)=X\wedge X\neq \emptyset \right\}\right). \end{array}$$

We give an example to show that *C*(*R*) is defined through the lower approximation based on a tolerance relation.

**Example 15 .**

Let *U* = {1,2, 3,4} and *R* = {(1,1), (2,2), (3,3), (4,4), (1,2), (2,1), (2,4), (4,2)} be a tolerance relation on *U*. Then we can get *RN*(1) = {1,2}, *RN*(2) = {1,2, 4}, *RN*(3) = {3}, and *RN*(4) = {2,4}. Then $\underline{R}(\{1,2,4\})=\{1,2,4\}$, $\underline{R}(\{3\})=\{3\}$, and $\underline{R}(\{1,2,3,4\})=\{1,2,3,4\}$. So *C*(*R*) = {{1,2, 4}, {3}}.

In the following proposition, we will prove *C*(*R*) to satisfy the circuit axioms of matroids when the relation is a tolerance relation.

**Proposition 16 .**

Let *R* be a tolerance relation on *U*. Then *C*(*R*) satisfies (C1), (C2), and (C3) of Proposition 10.

**Proof**

(1) From the definition of *C*(*R*), it is clear that *∅* ∉ *C*(*R*).

(2) Let *C*_1_, *C*_2_ ∈ *C*(*R*) and *C*_1_⊆*C*_2_. Because the elements of *C*(*R*) are minimal, we can get *C*_1_ = *C*_2_.

(3) Let *C*_*i*_, *C*_*j*_ ∈ *C*(*R*) and *C*_*i*_ ≠ *C*_*j*_. Then $\underline{R}(C_{i})=C_{i}$, $\underline{R}(C_{j})=C_{j}$. Suppose *C*_*i*_∩*C*_*j*_ ≠ *∅* and let *X* = *C*_*i*_∩*C*_*j*_. $\underline{R}(X)=\underline{R}(C_{i}\cap C_{j})=\underline{R}(C_{i})\cap \underline{R}(C_{j})=C_{i}\cap C_{j}=X$; then *X* ∈ *C*(*R*). Because *C*_*i*_, *C*_*j*_ are minimal elements, it is contradictory to the definition of *C*(*R*). Then *X* = *∅*. So for any *C*_*i*_, *C*_*j*_ ∈ *C*(*R*) and *C*_*i*_ ≠ *C*_*j*_, *C*_*i*_∩*C*_*j*_ = *∅*. Hence *C*(*R*) satisfies (*C*3).

In sum, this completes the proof.

A matroid and its circuits determine each other. Therefore, *C*(*R*) can generate a matroid when the relation is a tolerance relation.

**Definition 17 .**

Let *R* be a tolerance relation on *U*. The matroid with *C*(*R*) as its circuit family is denoted by *M*(*R*) = (*U*, *I*(*R*)), where *I*(*R*) = Opp(Upp(*C*(*R*))). One calls *M*(*R*) the matroid induced by *R*.

**Example 18 (continued from Example 15).**

Because *C*(*R*) = {{1,2, 4}, {3}}, we can get a matroid *M*(*R*) = (*U*, *I*(*R*)), where *I*(*R*) = {*∅*, {1}, {2}, {4}, {1,2}, {1,4}, {2,4}}.

In order to further understand this type of matroids, we introduce a special matroid called partition-circuit matroid.

**Definition 19 (partition-circuit matroid [34]).**

Let *M* = (*U*, *I*) be a matroid. If *C*(*M*) is a partition of *U*, *M* is called a partition-circuit matroid.

As shown in Proposition 16, we can prove that the matroid based on a tolerance relation is a partition-circuit matroid.

**Proposition 20 .**

Let *R* be a tolerance relation on *U*. The matroid *M*(*R*) is a partition-circuit matroid.

**Proof**

According to the proof of Proposition 16, we have known, for any *C*_*i*_, *C*_*j*_ ∈ *C*(*R*), *C*_*i*_∩*C*_*j*_ = *∅*. Because ${⋃}_{}C_{i}={⋃}_{}\underline{R}(C_{i})\subseteq \underline{R}({⋃}_{}C_{i})\subseteq {⋃}_{}C_{i}$ for all *C*_*i*_ ∈ *C*(*R*), $\underline{R}({⋃}_{}C_{i})={⋃}_{}C_{i}$. Suppose ⋃*C*_*i*_ ≠ *U*. Let *Y* = *U* − ⋃*C*_*i*_. Namely, *Y* ∉ *C*(*R*). Since $\underline{R}(Y)\neq Y$, there exists *y* ∈ *Y* such that *RN*(*y*)⊈*Y*. That is to say, there exists *x* ∈ *U* − *Y* = ⋃*C*_*i*_ such that *x* ∈ *RN*(*y*). Because *R* is a symmetric relation, *y* ∈ *RN*(*x*). It is contradictory to $\underline{R}({⋃}_{}C_{i})={⋃}_{}C_{i}$. Then ⋃*C*_*i*_ = *U*. Therefore, *C*(*R*) is a partition of *U*. This implies that *M*(*R*) is a partition-circuit matroid.

Transitive closure of a relation is an important concept for rough sets and matroids. We give the definition of transitive closure of a relation as follows.

**Definition 21 (transitive closure [35]).**

Let *R* be a relation on *U*. The smallest transitive relation on *U* containing the relation *R* is called the transitive closure of *R*. One denotes the transitive closure of *R* by *t*(*R*).

We give the properties of the corresponding transitive closure *t*(*R*) when *R* is a tolerance relation in the following lemma.

**Lemma 22 (see [36]).**

Let *R* be a tolerance relation on *U*. *t*(*R*) is an equivalence relation.

In [35], we can get *t*(*R*) = *R* ∪ *R* ^2^ ∪ ⋯. We know *t*(*R*) is an equivalence relation if *R* is a tolerance relation on *U*, so we can get a partition *U*/*t*(*R*) = {*P*_1_, *P*_2_,…, *P*_*m*_} on *U*, where *P*_1_, *P*_2_,…, *P*_*m*_ are the equivalence classes. Firstly, in order to show the connection between *C*(*R*) and the partition induced by the transitive closure of the tolerance relation, we give a lemma as follows.

**Lemma 23 (see [36]).**

Let *R*_*i*_  (*i* = 1,2,…) be relations on *U*. Then $\underline{{⋃}_{i=1}^{\infty }R_{i}}(X)={⋂}_{i=1}^{\infty }{\underline{R}}_{i}(X)$ for any *X*⊆*U*. $\underline{R^{n}}(X)={\underline{R}}^{\left(n\right)}(X)$ for any *X*⊆*U*, *n* = 1,2,…, where ${\underline{R}}^{\left(n\right)}$ stands for n-times composition of mapping $\underline{R}$.

Using the above lemmas, we can obtain an important proposition in the following.

**Proposition 24 .**

Let *R* be a tolerance relation on *U*. Then *C*(*R*) = *U*/*t*(*R*).

**Proof**

For any *X* ∈ *C*(*R*), $\underline{R}(X)=X$, $\underline{t(R)}(X)=\underline{{⋃}_{i=1}^{\infty }R^{n}}(X)={⋂}_{i=1}^{\infty }{\underline{R}}^{n}(X)={⋂}_{i=1}^{\infty }{\underline{R}}^{\left(n\right)}(X)=\underline{R}(X)\cap {\underline{R}}^{\left(2\right)}(X)\cap \cdots$. Since $\underline{R}(X)=X$, $\underline{t(R)}(X)=X$. Because *t*(*R*) is an equivalence relation, *X* ∈ *U*/*t*(*R*). Therefore, *C*(*R*)⊆*U*/*t*(*R*). Conversely, for all *X* ∈ *U*/*t*(*R*). Since *t*(*R*) is an equivalence relation, $\underline{t(R)}(X)=X$. Because *t*(*R*) is also a tolerance relation, *X* ∈ *C*(*R*). This proves *U*/*t*(*R*)⊆*C*(*R*). In sum, this completes the proof.

An example can illustrate that *C*(*R*) is equal to the partition induced by the transitive closure of the tolerance relation.

**Example 25 (continued from Example 15).**

We know that *U* = {1,2, 3,4}, *R* = {(1,1),  (2,2),  (3,3),  (4,4),  (1,2),  (2,1),  (2,4),  (4,2)}, and *C*(*R*) = {{1,2, 4}, {3}}. So the transitive closure *t*(*R*) = {(1,1), (2,2), (3,3), (4,4), (1,2), (2,1), (2,4), (4,2), (1,4),(4,1)}. Therefore, *U*/*t*(*R*) = {{1,2, 4}, {3}}. It is clear that *C*(*R*) = *U*/*t*(*R*).

In [34], Liu has already shown the characteristics of independent sets about partition-circuit matroid. Combining the results of Liu about partition-circuit matroid, we can get the expression of the independent sets of the matroid induced by a tolerance relation.

**Lemma 26 (see [34]).**

Let *R* be a tolerance relation on *U*. *M*(*R*) is the matroid induced by *R*. Then,

(5) $$\begin{array}{c} I\left(R\right)=\left\{X\subseteq U:\forall C\in C\left(R\right),\left|X\cap C\right|\leq \left|C\right|-1\right\}. \end{array}$$

We present the expression of the base according to the partition induced by the transitive closure of a tolerance relation.

**Proposition 27 .**

Let *R* be a tolerance relation on *U*. *M*(*R*) is the matroid induced by *R*. Then,

(6) $$\begin{array}{c} B\left(R\right)=\left\{X\subseteq U:\forall P\in \frac{U}{t\left(R\right)},\left|X\cap P\right|=\left|P\right|-1\right\}. \end{array}$$

**Proof**

Because *B*(*R*) = *Max*⁡(*I*(*R*)) and *I*(*R*) = {*X*⊆*U* : for  all  *C* ∈ *C*(*R*), |*X*∩*C*| ≤ |*C*| − 1}, *B*(*R*) = {*X*⊆*U* : for  all  *C* ∈ *C*(*R*), |*X*∩*C*| = |*C*| − 1}. Based on Proposition 24, *B*(*R*) = {*X*⊆*U* : for  all  *P* ∈ *U*/*t*(*R*), |*X*∩*P*| = |*P*| − 1}.

The rank function of the matroid induced by a tolerance relation can be well expressed by the the partition induced by the transitive closure of the tolerance relation.

**Proposition 28 .**

Let *R* be a tolerance relation on *U*. *M*(*R*) is the matroid induced by *R*. Then, for all *X*⊆*U*,

(7) $$\begin{array}{c} r_{M(R)}\left(X\right)=\left|X\right|-\left|\left\{P\in \frac{U}{t\left(R\right)}:P\subseteq X\right\}\right|. \end{array}$$

**Proof**

According to Proposition 12, we need to prove *r*_*M*(*R*)_(*X*) = |*X*| if and only if *X* ∈ *I*(*R*). When *r*_*M*(*R*)_(*X*) = |*X*|, |{*P* ∈ *U*/*t*(*R*) : *P*⊆*X*}| = 0; namely, |{*C* ∈ *C*(*R*) : *C*⊆*X*}| = 0, based on Proposition 24. So for any *C* ∈ *C*(*R*), |*X*∩*C*| ≤ |*C*| − 1. Hence, *X* ∈ *I*(*R*). Conversely, *X* ∈ *I*(*R*) and for any *C* ∈ *C*(*R*), |*X*∩*C*| ≤ |*C*| − 1. Because *C*(*R*) = *U*/*t*(*R*), for any *P* ∈ *U*/*t*(*R*), |*X*∩*P*| ≤ |*P*| − 1. Therefore, *X* ≠ *P* and *P*⊈*X*. So |{*P* ∈ *U*/*t*(*R*) : *P*⊆*X*}| = 0. Therefore *r*_*M*(*R*)_(*X*) = |*X*|. In sum, this completes the proof.

In order to better illustrate the feature of the rank function, we give the rank for all subsets of universe.

**Example 29 (continued from Example 15).**

We have known *C*(*R*) = {{1,2, 4}, {3}} and *U*/*t*(*R*) = {{1,2, 4}, {3}}. Suppose *X* = {1,2}. There does not exist *P* ∈ *U*/*t*(*R*) such that *P*⊆*X*. Then *r*_*M*(*R*)_(*X*) = |*X*| = 2. Suppose *X* = {1,3}. There exists a *P* = {3} ∈ *U*/*t*(*R*) such that *P* = {3}⊆*X*. Therefore, *r*_*M*(*R*)_(*X*) = |*X*| − 1 = 1.

Rough set theory and matroid theory have close relationships. We study the connection between the closure of the matroid induced by a tolerance relation and the upper approximation of the tolerance relation when *C*(*R*) does not contain any single-point set.

**Proposition 30 .**

Let *R* be a tolerance relation on *U*. If *C*(*R*) does not contain any single-point set, then ${\mathrm{cl}}_{M(R)}(X)\subseteq \bar{R}(X)$ for any *X*⊆*U*.

**Proof**

Since cl⁡_*M*(*R*)_(*X*) = *X* ∪ {*e* ∈ *U* − *X* : ∃*C* ∈ *C*(*R*) such that *e* ∈ *C*⊆*X* ∪ {*e*}}, we need to prove {*e* ∈ *U* − *X* : ∃*C* ∈ *C*(*R*) such that $e\in C\subseteq X\cup \{e\}\}\subseteq \bar{R}(X)$. That is to say, for any *e* ∈ *U* − *X*, there exists *C* ∈ *C*(*R*) such that *e* ∈ *C*⊆*X* ∪ {*e*}. We can get *RN*(*e*)∩*X* ≠ *∅*. If *RN*(*e*)∩*X* = *∅*, then there exist two different circuits such that *RN*(*e*) and *X* are contained in them, respectively. If there exists only one circuit *C* ∈ *C*(*R*) such that *RN*(*e*)⊆*C* and *X*⊆*C*, then *e* ∈ *RN*(*e*)⊆*C*⊆*X* ∪ {*e*}⊆*C* ∪ {*e*}. Because *C*(*R*) does not contain any single-point set, |*C*| ≥ 2. It is a contradiction. So there exist two different circuits *C*_1_ ∈ *C*(*R*) and *C*_2_ ∈ *C*(*R*) such that *RN*(*e*)⊆*C*_1_ and *X*⊆*C*_2_. Then *e* ∈ *RN*(*e*)⊆*C*_1_⊆*X* ∪ {*e*}⊆*C*_2_ ∪ {*e*}. Since *C*_1_∩*C*_2_ = *∅* and |*C*_*i*_| ≥ 2  (*i* = 1,2), *C*_1_⊆*C*_2_ ∪ {*e*} does not hold. So *RN*(*e*)∩*X* ≠ *∅*. That is to say, $e\in \bar{R}(X)$. Hence, ${\mathrm{cl}}_{M(R)}(X)\subseteq \bar{R}(X)$.

But if *C*(*R*) contains single-point sets, in general, ${\mathrm{cl}}_{M(R)}(X)\subseteq \bar{R}(X)$ does not hold. We can use an example to illustrate this situation.

**Example 31 (continued from Example 15).**

We have known *C*(*R*) = {{1,2, 4}, {3}} and the transitive closure *t*(*R*) = {(1,1), (2,2), (3,3), (4,4), (1,2), (2,1), (2,4), (4,2), (1,4),(4,1)}. If *X* = {1}, we can get cl⁡_*M*(*R*)_({1}) = {1,3}, $\bar{R}(\{1\})=\{1,2\}$. There is no relationship between $\bar{R}(X)$ and cl⁡_*M*(*R*)_(*X*).

We do not consider single-point sets of *C*(*R*) in the above proposition. If *C*(*R*) contains single-point sets, we show the relationship between the the upper approximation of a tolerance relation and the closure of the matroid induced by the tolerance relation.

**Corollary 32 .**

Let *R* be a tolerance relation on *U*. For any *X*⊆*U*, ${\mathrm{cl}}_{M(R)}(X)-\{x\in U:\{x\}\in C(R)\}\subseteq \bar{R}(X)$.

For any *X* ∈ *C*(*R*), we have the following conclusion about $\bar{t(R)}(X)$, $\bar{R}(X)$, and cl⁡_*M*(*R*)_(*X*).

**Proposition 33 .**

Let *R* be a tolerance relation on *U*. For all *X* ∈ *C*(*R*), $\bar{t(R)}(X)=\bar{R}(X)\subseteq {\mathrm{cl}}_{M(R)}(X)$.

**Proof**

For any *X* ∈ *C*(*R*), $\underline{R}(X)=X$. Since *R* is a symmetric relation, $\bar{R}(\underline{R}(X))\subseteq X$. Therefore, we can get $\bar{R}(X)\subseteq X$. *R* is also a reflexive relation, so $X\subseteq \bar{R}(X)$. Thus $\bar{R}(X)=X$. Since *X*⊆cl⁡_*M*(*R*)_(*X*), we can get $\bar{R}(X)\subseteq {\mathrm{cl}}_{M(R)}(X)$ for all *X* ∈ *C*(*R*). Because *C*(*R*) = *U*/*t*(*R*), for any *X* ∈ *C*(*R*), *X* ∈ *U*/*t*(*R*). Then $\bar{t(R)}(X)=X=\bar{R}(X)$. So $\bar{t(R)}(X)=X\subseteq {\mathrm{cl}}_{M(R)}(X)$. Hence $\bar{t(R)}(X)=\bar{R}(X)\subseteq {\mathrm{cl}}_{M(R)}(X)$.

We have discussed how to induce a matroid from a relation. Then, how to induce a relation from a matroid is presented as follows.

**Definition 34 (see [17]).**

Let *M* = (*U*, *I*) be a matroid. We define a relation *R*(*M*) on *U* as follows: for all *x*, *y* ∈ *U*,

(8) (x,y)∈R(M)⟺x=y or ∃C∈C(M)such  that  {x,y}⊆C.

We say *R*(*M*) is a relation on *U* induced by *M*.

An example is provided to illustrate how to induce a relation from a matroid.

**Example 35 .**

Let *M* = (*U*, *I*) be a matroid, where *U* = {1,2, 3,4} and *I* = {*∅*, {1}, {2}, {3}, {1,2}, {1,3}, {2,3}}. Since *C*(*M*) = {{1,2, 3}, {4}}, *R*(*M*) = {(1,1), (2,2), (3,3), (4,4), (1,2), (2,1), (1,3), (3,1), (2,3),(3,2)}.

According to the above definition, any matroid can induce a relation. The following lemma proves that the relation induced by a matroid is an equivalence relation.

**Lemma 36 (see [17]).**

Let *M* = (*U*, *I*) be a matroid and *R*(*M*) the relation induced by *M*. Then *R*(*M*) is an equivalence relation on *U*.

We know a tolerance relation can induce a matroid and the matroid can also induce a relation. In the following proposition, we give the relationship between the original tolerance relation and the induced relation.

**Proposition 37 .**

Let *R* be a tolerance relation on *U*. Then *R*⊆*R*(*M*(*R*)).

**Proof**

Let (*x*, *y*) ∈ *R*. Because *R* is a reflexive relation, (*x*, *x*) ∈ *R* and (*y*, *y*) ∈ *R*. *R* is also a symmetric relation, so (*y*, *x*) ∈ *R*. Thus {*x*, *y*}⊆*RN*(*x*) and {*x*, *y*}⊆*RN*(*y*). So there exists *C* ∈ *C*(*R*) such that *RN*(*x*)⊆*C* and *RN*(*y*)⊆*C*; that is, {*x*, *y*}⊆*C*. Therefore (*x*, *y*) ∈ *R*(*M*(*R*)). Hence *R*⊆*R*(*M*(*R*)).

In the above proposition, we give the connection between the original tolerance relation and the induced relation, while we give the relationship between the induced relation and the transitive closure of the original tolerance relation in the following proposition.

**Proposition 38 .**

Let *R* be a tolerance relation on *U*. Then *t*(*R*) = *R*(*M*(*R*)).

**Proof**

For any (*x*, *y*) ∈ *R*(*M*(*R*)), *x* = *y* or ∃*C* ∈ *C*(*M*) such that {*x*, *y*}⊆*C*. Suppose *x* = *y*. Since *t*(*R*) is an equivalence relation, (*x*, *y*) ∈ *t*(*R*). Suppose there exists *C* ∈ *C*(*M*) such that {*x*, *y*}⊆*C*. That is to say, there exists *P* ∈ *U*/*t*(*R*) such that {*x*, *y*}⊆*P*. So (*x*, *y*) ∈ *t*(*R*). Therefore, *R*(*M*(*R*))⊆*t*(*R*). Because *t*(*R*) is the smallest transitive relation containing *R*, *t*(*R*) is an equivalence relation and *R*(*M*(*R*)) is also an equivalence relation: *t*(*R*) = *R*(*M*(*R*)).

## 4. Conclusions

In this paper, we connected matroid theory and generalized rough set theory based on relations. We firstly defined a family of sets induced by a tolerance relation and proved the family to satisfy the circuit axioms of matroids. Some characteristics of this matroid, such as the base and the rank function, were studied. Then, we investigated the relationship between the upper approximation of a tolerance relation and the closure operator of the matroid induced by the tolerance relation. Finally, the matroid established by a tolerance relation could induce a relation. We studied the connection between the original tolerance relation and the induced relation. This study provides an important connection between generalized rough sets based on relations and matroids.
